# Evaluation of the Effects of Various Types of Mouthwash on Enamel and Cementum of Permanent Teeth: An In Vitro Study

**DOI:** 10.1155/tswj/5583978

**Published:** 2026-03-31

**Authors:** Mita Shanbhag, Amitha J. Lewis, N. Srikant

**Affiliations:** ^1^ Manipal College of Dental Sciences Mangalore, Manipal Academy of Higher Education, Manipal, Mangalore, Karnataka, India, manipal.edu; ^2^ Department of Oral Pathology and Microbiology, Manipal College of Dental Sciences Mangalore, Manipal Academy of Higher Education, Manipal, Mangalore, Karnataka, India, manipal.edu

**Keywords:** cementum surface hardness, enamel, mouthwash, roughness

## Abstract

**Background:**

Mouthwashes are widely utilised for their affordability, ease of use and oral hygiene benefits. However, indiscriminate use of these over‐the‐counter mouthwashes can cause morphological changes in enamel and cementum, compromising tooth structure. This study evaluates the change in the surface roughness, surface hardness and colour of enamel and cementum after the use of various mouthwashes using surface profilometry, Vickers hardness tester and spectrophotometry, respectively, for 7 and 15 days.

**Materials and Methodology:**

Five common mouthwashes, namely, Colgate Plax, Listerine, Himalaya, Chlorhexidine and warm saline water were tested on 10 extracted permanent teeth in each group. Teeth were sectioned longitudinally from crown to root, embedded in acrylic and examined for defects. Baseline values for colour, hardness and roughness were recorded before immersing the samples in mouthwash as per the manufacturers’ instructions. Postimmersion measurements were taken at specified intervals.

**Results:**

A significant decrease in enamel hardness after 7 days with chlorhexidine (108.28 ± 85.17), Himalaya (104.02 ± 94.22), warm saline (85.42 ± 70.9) and Listerine (55.32 ± 83.68) was observed, but not with Colgate Plax. Cementum hardness decreased over 15 days but was not statistically significant. The greatest increase in surface roughness was seen in saline followed by Himalaya whereas the decrease was seen in chlorhexidine and Colgate Plax at the end of 7 days. Listerine caused the highest colour change in enamel. Since trends towards decreased hardness and increased roughness were noted, long‐term usage could pose clinical concerns. Warm saline showed subtle yet significant changes, indicating the need for further investigation.

**Conclusion:**

These findings underscore the importance of regulating mouthwash use and recommending it only when the benefits outweigh the risks.

## 1. Introduction

Control of biofilm is of paramount importance in preventive dentistry as it directly affects the oral health of an individual. The biofilm houses pathogenic microorganisms responsible for dental caries and periodontal diseases. Adequate control of this biofilm can be achieved through mechanical methods like tooth brushing and flossing, chemical agents like mouthwashes/mouth rinses or a combination of both [1]. Mouth rinses serve as valuable adjuncts in oral health care, widely utilised for their affordability, user‐friendliness and pleasant taste. They effectively reduce halitosis, penetrate plaque and retain adequately at disease sites, reaching less accessible areas. Additionally, their stable storage, remineralising capabilities and antimicrobial properties significantly contribute to the maintenance of dental structure and hygiene [2, 3]. Mouth rinses are specially prescribed by dentists for patients with gingival and periodontal diseases wherein the mechanical methods alone are not adequate to prevent the recurrence and progression of disease [4].

0.12% chlorhexidine gluconate (CHX) has been considered the gold standard for antimicrobial mouth rinses. It has a pH range of 5.5–7 and an alcohol content of 11.6% as per the manufacturers of CHX. It can alter the bacterial cell wall and lead to rupture of the cell. It binds to salivary mucins and reduces pellicle formation. However, long‐term use of chlorhexidine can cause staining of the teeth. Hence, it needs to be prescribed by a medical/dental practitioner [5].

However, presently mouthwashes are easily accessible as over‐the‐counter products. They are readily available in supermarkets and drugstores and can be ordered through e‐commerce platforms [2]. Among these, mouth rinses containing essential oils (EOs) and cetylpyridinium chloride (CPC) are also present. The mouth rinses containing EOs are found to be as effective as chlorhexidine and contain eucalyptol, methyl salicylate, thymol as well as alcohol that may vary from 20% to 27%. The mouth rinses act by rapidly disrupting the enzymes that form the biofilm and by reducing the endotoxin levels and pathogenicity of plaque [5].

In contrast, CPC mouth rinses, though they have a broad antimicrobial activity, their effect is said to be lesser than chlorhexidine and EOs in controlling plaque and gingivitis. CPC acts by its action on the bacterial cell wall, inhibiting cell growth and bacterial metabolism.

Low pH of carbonated beverages has been shown to cause a change in the surface characteristics of enamel and cementum [6]. Though mouth rinses are beneficial for the reduction of plaque and gingivitis, literature shows that many of the mouth rinses have a low pH [5, 7]. In the absence of guidance from a professional, indiscriminate use of mouthwashes that expose the oral cavity to these substances for an extended period may produce undesired morphological changes in enamel, ultimately compromising the tooth structure [3]. The effects of mouth rinse on enamel are still controversial. Though a few studies have been conducted, some report that continuous use can cause changes on the surface of enamel [2, 3] whereas other authors did not find any significant changes in the tooth enamel subjected to these mouth rinses for immersion period simulating the regular use [1].

Every Indian home has a warm saline water gargle as a go‐to cure for a range of ailments. It is also highly recommended after dental procedures due to its multitude of benefits such as pH alteration causing a reduction in microbial activity and promoting human gingival fibroblast wound healing [8].

The goal of this study was to comprehend the impact of mouthwashes on dental structure. Thus, we conducted a time‐based objective study using the commonly used mouth rinses and warm saline to evaluate the change in surface characteristics, namely, microhardness and surface roughness of enamel and cementum and colour of enamel.

## 2. Materials and Methods

The present study was conducted at our Institution in association with other neighbouring institutes. The study commenced only after obtaining clearance from the Institutional Ethics Committee.

Commonly used mouthwashes, namely, Colgate Plax, Listerine, Himalaya and Chlorhexidine, were used as test samples and warm saline water was used as a control. The warm saline water was prepared by mixing about 1 teaspoon of salt with 100 mL of water. The pH of each of the mouthwashes was checked prior to exposure of the teeth with a pH meter (Elico LI 615 pH meter) connected to an electrode calibrated with standard solutions of pH 4.0 and 9.0, respectively. The pH value was recorded when the reading on the meter was stable (Table 1). Between each of the measurements, the probe was rinsed in deionised water. The readings were carried out at room temperature.

**Table 1:** pH of the solutions.

**Solutions**	**pH values**	**Key ingredients**
Listerine cavity fighter	4.5	Eucalyptol, methyl salicylate, thymol, menthol and sodium fluoride.
Colgate Plax (active salt)	5.8	Water, glycerin, propylene glycol, sorbitol, sodium chloride, aroma, sodium saccharin, Polysorbate 20, aroma, cetylpyridinium chloride (CPC), potassium sorbate, sodium fluoride, menthol, *Lonicera japonica* flower extract, Cl 17200, CI 19140, CI 42051, Poloxamer 407
Chlorhexidine	5.9	0.12% chlorhexidine gluconate in a base containing water, 11.6% alcohol, glycerin, PEG‐40 sorbitan diisostearate, flavor, sodium saccharin and FD&C Blue No.1.
Himalaya HiOra‐K (for sensitive teeth)	5.1	Clove, potassium nitrate
Warm saline	7.1	Water, salt

### 2.1. Sample Size Calculation

Based on the key article titled, “Effect of Mouth Rinses on Tooth Enamel Surface” and basing our values on the parameter of microhardness as given in the table, having four groups with a power of 80% and an alpha error rate of 5% the *Z* score are 0.841621233572915 and 3.12373463032385, respectively, the highest standard deviation of 5.53 and to assess a clinically relevant difference (*d*) of 10, we need a sample of eight per group, using the formula $N=\left[2{\left(Z_{12-a/*k}+Z_{1-}\beta \right)}^{2}\sigma^{2}\right]/d^{2}$ where sigma is the standard deviation and *d* is the clinically significant difference. To account for 10% errors in tooth or measurement errors, we took a sample of 10 in each group.

### 2.2. Preparation of Tooth Samples

Fifty extracted permanent teeth were carefully cleaned and stored in distilled water. Teeth without caries and enamel defects and those primarily extracted for orthodontic/therapeutic purposes were included in the study. The teeth that did not meet the inclusion criteria were excluded from the study. The teeth were sectioned longitudinally into two parts. Each of the halves was embedded into acrylic taking care that the buccal/lingual surface faces upwards to provide a firm base for the usage of the atomic force microscope and the Vickers hardness tester. The prepared specimens were observed under a stereomicroscope to check for any cracks or surface defects. Each specimen was assigned a number. The prepared samples were then stored in distilled water to avoid dehydration.

### 2.3. Baseline Measurement

Surface hardness evaluation: The baseline surface microhardness of all the samples was determined using a digital micro‐Vickers hardness tester (Matsuzawa Model: MMT‐X7A) with a diamond indenter. The indentation load was 100 g with a dwell time of 10 s. Three indentations each were performed on the crown and root of each tooth sample.

Surface roughness evaluation: The Ra surface roughness (mean surface roughness) of the samples was found using an atomic force microscope (Flex‐Axiom AFM, Nanosurf, Switzerland). An area of 20 × 20 *μ*m was measured by tapping method in the crown and root at the speed of 1 mHz on each sample. The mean of the area was measured with the help of Gwyddion software, and those values were used in the statistical analysis. Each sample was measured at three points in time: baseline and Day 7 of mouth rinse application.

Baseline colour evaluation: The specimens were dried with the help of an absorbent paper before colour evaluation. A photograph was taken with the specimen placed on a grey shade‐card. The photograph was then opened through ImageJ software. The images were then converted by slitting the three layers of *L*, *a* and *b*. A square of 30 pixels was then drawn using the rectangular selection tool, which was placed on top of the grey shade card and measured for the intensity of *L*, *a* and *b* values. The same 30 square pixel area was used for measuring the intensity on the enamel and cementum. These values were then corrected for the grey values and calculated for absolute values of *L*, *a* and *b*. The value *L* represents the degree of lightness which varies from black (0) to white (100). The value *a* ^∗^ and *b* ^∗^ denotes the red (+*a* ^∗^), green (−*a* ^∗^), yellow (+*b* ^∗^) and blue (−*b* ^∗^) degree in the specimens [2] The differences were then calculated as *Δ* *L*, *Δ* *a* and *Δ* *b*. Then, the extent of the colour change (*Δ* *E*) was assessed using the following equation, ΔE=L12−L2+a12−a2+  b12−b2.

### 2.4. Immersion in Mouthwashes

The specimens were immersed in mouthwashes after brushing them with fluoridated toothpaste for 1 min according to the manufacturer’s instructions.

#### 2.4.1. Listerine Cavity Fighter

Rinse undiluted, twice a day for 30 s. Do not rinse mouth with water or eat and drink anything for 30 min postrinse.

#### 2.4.2. Colgate Plax (Active Salt) or Complete Care

Rinse two times a day after brushing for 30 s.

#### 2.4.3. Chlorhexidine

Swish in the mouth for 1 min. Do not wash mouth for 1 min. Do not wash mouth or eat thereafter for 20 min.

#### 2.4.4. Himalaya HiOra‐K (for Sensitive Teeth)

Rinse mouth thoroughly for 30 s and expel. Use twice daily.

#### 2.4.5. Warm Saline Rinse

Rinse two times a day. All the exposures were carried out with constant agitation in a beaker at 37°C to simulate the oral procedure. This procedure was repeated twice per day. The specimens were stored in distilled water in between the immersion intervals. The surface hardness and colour of enamel, enamel and cementum were measured at an interval of 7 and 15 days, whereas the surface roughness was measured at an interval of 7 days. The measurements taken at each interval were noted.

### 2.5. Statistics

The differences in colour, surface hardness and roughness caused by each of the mouthwashes were compared using one‐way ANOVA and post hoc Tukey test. The difference in hardness and roughness between the periods of immersion were analysed using the *t*‐test.

## 3. Observations and Results

The mean baseline of Vicker’s hardness for enamel was 265.64 (82.83) for permanent teeth. At the end of 7 days, enamel hardness decreased after immersion in all mouth rinses except Colgate Plax. The greatest decrease in hardness was seen in enamel samples placed in chlorhexidine (108.28 ± 85.17) followed by Himalaya (104.02 ± 94.22), warm saline (85.42 ± 70.9) and Listerine (55.32 ± 83.68). However, the decrease was statistically significant only in chlorhexidine and warm saline. Overall, at the end of 15 days, the trend towards a decrease in microhardness was observed in enamel placed in all the mouth rinses; however, it was not statistically significant (Table 2). The mean baseline hardness of cementum was found to be 47.06 (20.79). The hardness of cementum decreased at the end of 7 days and continued till the end of 15 days. However, this was not statistically significant (Table 2).

**Table 2:** Surface hardness values compared between groups.

	**Colgate Plax (** **n** = 10**)**	**Listerine whitening (** **n** = 10**)**	**Himalaya (** **n** = 10**)**	**Chlorhexidine (** **n** = 10**)**	**Warm saline (** **n** = 10**)**	**F** **value ANOVA**	**p** **value**
Enamel baseline hardness	228.54 ± 104.77	239.27 ± 76.7	303.44 ± 83.41	297 ± 64.4	259.95 ± 66.18	1.745	0.157
Enamel 7 days hardness	251.28 ± 77.17^*π*^	183.95 ± 31.89	199.42 ± 24.25	188.72 ± 60.45	174.53 ± 40.08^*π*^	2.148	0.109
Enamel hardness difference 7 days	−22.74 ± 87.32^*π*,*α*,*μ*^	55.32 ± 83.68	104.02 ± 94.22^*α*^	108.28 ± 85.17^*μ*^	85.42 ± 70.9^*π*^	4.052	**0.007**
*t*(*p* value): paired *t*‐test	−0.823 (0.432)	2.091 (0.066)	3.491 (0.007)	**4.02 (0.003)**	**3.81 (0.004)**		
Cementum baseline hardness	44 ± 19.19	45.98 ± 22.35	57.99 ± 29.86	42.58 ± 12.55	44.76 ± 16.28	0.89	0.478
Cementum 7 days hardness	35.53 ± 8.73	30.92 ± 10.09	45.04 ± 18.13	33.26 ± 8.65	31.67 ± 9.35	1.286	0.305
Cementum hardness difference 7 days	8.47 ± 21.39	15.06 ± 22.18	12.95 ± 33.47	9.32 ± 16.62	13.09 ± 13.75	0.152	0.961
*t*(*p* value): paired *t*‐test	1.252 (0.242)	2.147 (0.06)	1.223 (0.252)	1.773 (0.11)	**3.01 (0.015)**		

There was no significant difference in the surface roughness of enamel and cementum at baseline or 7 days. On comparison of the difference in the roughness of enamel, it was found that Listerine, Himalaya and warm saline showed an increase in surface roughness. The highest roughness was seen in saline 95.53 units, followed by Himalaya 57.98, followed by Listerine 36.7 units. Chlorhexidine showed the highest decrease in surface roughness with 87.21 units followed by Colgate Plax, which showed a 25.75 units decrease in surface roughness. However, the differences in roughness were not significant among the five groups. Analysis of cementum showed an increase in surface roughness in all mouthwashes except Listerine. The highest roughness was noted with saline, followed by Himalaya. Listerine, which had shown an increase in roughness in enamel, showed a decrease in roughness in cementum. However, the change was not statistically significant in any analysis (Table 3).

**Table 3:** Surface roughness values compared between the groups.

	**Colgate Plax (** **n** = 10**)**	**Listerine (** **n** = 10**)**	**Himalaya (** **n** = 10**)**	**Chlorhexidine (** **n** = 10**)**	**Saline (** **n** = 10**)**	**Statistic/** **F**	**p** **value**
Enamel baseline	210.29 ± 118.57	169.81 ± 58.03	185.99 ± 130.17	364.28 ± 194.36	158.12 ± 71.45	2.497	0.073
Enamel Day 7	184.54 ± 90.62	206.51 ± 189.32	243.97 ± 94.83	277.02 ± 131.05	253.65 ± 223.07	0.577	0.681
Enamel difference	−25.75 ± 141.14	36.7 ± 174.35	57.98 ± 157.82	−87.26 ± 161.8	95.53 ± 190.66	1.898	0.127
*t*(*p* value): paired *t*‐test	0.58 (0.578)	−0.67 (0.522)	−1.16 (0.275)	1.71 (0.122)	−1.58 (0.148)		
Cementum baseline	145.98 ± 49.21	196.25 ± 81.37	224.25 ± 122.45	266.44 ± 104.26	233.09 ± 90.88	2.362	0.067
Cementum Day 7	161.67 ± 110.33	172.99 ± 40.22	261.18 ± 137.3	284.96 ± 156.24	332.27 ± 551.89	2.093	0.12
Cementum difference	15.69 ± 100.39	−23.26 ± 81.04	36.93 ± 219.55	13.49 ± 218.78	99.18 ± 571.35	0.381	0.82
*t*(*p* value): paired *t*‐test	−0.49 (0.633)	0.91 (0.388)	−0.53 (0.608)	−0.28 (0.784)	−0.55 (0.596)		

Table 4 describes the mean and the standard deviation of *Δ* *L*, *Δ* *a* and *Δ* *b* and *Δ* *E* values of enamel. The results show that the *Δ* *E* value is highest for chlorhexidine in 7 days but is highest for Listerine in 15 days. The main colour coordinate in each group was statistically equal to that of the baseline. Listerine, followed by saline, showed the highest variation in colour of enamel in 15 days.

**Table 4:** Colour changes in enamel.

	**Colgate Plax (** **n** = 10**)**	**Listerine (** **n** = 10**)**	**Himalaya (** **n** = 10**)**	**Chlorhexidine (** **n** = 10**)**	**Saline (** **n** = 10**)**	**Statistic/** **F**	**p** **value**
Enamel *Δ* *L* 7 days	10.88 ± 13.23	10.21 ± 19.1	9.24 ± 9.59	1.13 ± 20.32	11.13 ± 13.28	0.452	0.77
Enamel *Δ* *a* 7 days	−14.78 ± 118.01	−44.99 ± 147.76	−80.35 ± 98.37	−408.05 ± 1110.23	12.86 ± 142.72	1.058	0.401
Enamel *Δ* *b* 7 days	−23.16 ± 22.12	−24.79 ± 28.33	−11.59 ± 16.49	−12.24 ± 14.2	−5 ± 44.67	0.934	0.453
Enamel *Δ* *E* 7 days	81.74 ± 90.21	85.99 ± 133.54	83.44 ± 98.6	417.24 ± 1106.78	116.54 ± 88.32	0.422	0.791
Enamel *Δ* *L* 15 days	6.1 ± 7.33	8.52 ± 11.18	5.96 ± 11.58	8.61 ± 9.83	4.25 ± 8.2	0.364	0.833
Enamel *Δ* *a* 15 days	10.02 ± 90.51	1318.93 ± 4149.63	−44.8 ± 30.44	135.26 ± 222.64	−347.95 ± 2139.27	2.401	0.086
Enamel *Δ* *b* 15 days	9.76 ± 16.41	2.89 ± 9.12	12.42 ± 16.27	13.88 ± 17.2	22.93 ± 41.13	1.01	0.412
Enamel *Δ* *E* 15 days	60.77 ± 68.35	1528.73 ± 4068.89	53.04 ± 25.3	185.04 ± 180.39	1001.42 ± 1897.12	2.058	0.126

## 4. Discussion

The appearance of the teeth is of prime concern to many patients seeking dental treatment. Nowadays, people are increasingly aware of the methods of maintaining oral hygiene and it is not restricted to mechanical methods alone: chemical oral hygiene measures have been utilised in combination with tooth brushing and flossing. The attractive labels on the bottles containing mouthwashes with captions like ‘cavity fighter’, ‘for sensitive teeth’, ‘tooth whitening’ and the easy availability of these mouthwashes in supermarkets and drug stores make consumers buy and use them indiscriminately. Many of these individuals are not aware of the long‐term potential effects of these mouthwashes on the enamel [2, 4].

Mouth rinses are prescribed by dentists in patients with gingivitis and/or periodontitis to reduce gingival inflammation by effectively controlling the biofilm. In such cases, these mouth rinses are usually prescribed for a week to 15 days. In the present study, we evaluated the change in colour, surface microhardness and surface roughness of human enamel and cementum at baseline, 7 days and 15 days following immersion in mouth rinses. The mouth rinses selected for this study were from different brands with varied chemical compositions. While chlorhexidine was the standard, one with EOs, one with CPC, a herbal product and finally warm saline were included. Warm saline rinses are often advised in patients’ postextraction. It is known to reduce inflammation and promote healing. It is also used as a go‐to cure in Indian households for any tooth‐related problems. However, the effect of warm saline on the tooth structure has not been studied to date. The pH range of all the mouth rinses was tested and was found to be between 4.5 and 5.8., that is, all of them were of acidic pH. Listerine had the lowest pH of 4.5, while Himalaya, Colgate Plax and chlorhexidine had a pH of 5.1, 5.8 and 5.9, respectively (Table 1). As all the mouth rinses had an acidic pH, some amount of demineralisation was expected. However, only Listerine and Himalaya had a pH below the critical pH of enamel dissolution. Enamel hardness decreased after immersion in all mouth rinses and warm saline at the end of 7 days except Colgate Plax. However, a significant decrease in microhardness was found only in chlorhexidine and warm saline after 7 days. At the end of 15 days, though there was a trend towards a decrease in enamel hardness, it was not statistically significant. The decrease in microhardness was in accordance with the study conducted by Favaro et al. [2] and Huynh et al. [8]. However, the study conducted by Favaro et al. was carried out for 12 weeks in contrast to the present study which was for 15 days. This indicates that a short duration of a fortnight has a clinically significant change in hardness in all the mouthwashes. The study conducted by Huynh et al. [8] included mouthwash with hydrogen peroxide commonly used for whitening the teeth. However, contrary to the present results, Palino et al. showed that long‐term exposure simulating 90 and 180 days to alcohol‐containing mouthwashes did not cause any morphological, ultrastructural or biochemical change on human enamel and restorative materials. However, they used artificial saliva as a medium to place the enamel samples in between the immersions and they did not adhere to the daily usage protocol as per the manufacturer’s instructions which may have resulted in the contrary findings. In the present study, we followed the manufacturer’s instructions for each of the mouth rinses tested. Thus, the contact time of chlorhexidine with the tooth samples was 1 min, unlike 30 s in the case of the other mouth rinses. Also, after swishing, as per the manufacturer’s instructions, a 20‐min no rinse protocol was followed. This protocol is designed to improve the efficacy of the mouthwash. However, studies [10, 11] have reported that the practice of holding the beverage in the mouth increases the erosive potential. This risk of erosion may in turn be compounded by not washing or eating for 20 min as may have happened in the present study with chlorhexidine mouthwash. Enamel samples placed in Colgate Plax in the first 7 days of immersion did not show a reduction in microhardness. This could be attributed to the fact that prior to immersion the tooth samples were brushed with fluoride toothpaste. The activity of fluoride was enhanced by the pH of 5.8 and other ingredients of Colgate Plax to prevent a reduction in hardness. Listerine also had sodium fluoride but owing to the lower pH the benefit of maintaining the pH was possibly not obtained.

The surface roughness at the end of 7 days varied across each group. A maximum increase in surface roughness was seen in Listerine, followed by warm saline. Listerine also recorded the lowest pH range among all the groups. Thus, an increase in roughness can be expected. The results were in accordance with that by Favaro et al. [2] who also reported an increase in enamel surface roughness in enamel samples placed in different mouth rinses. Chlorhexidine and Colgate Plax usage showed a decrease in surface roughness at the end of 7 days. It could be possibly attributed to the formation of a solute layer that could have smoothened the enamel surface. This change in texture and composition alters the colour of the enamel surface.

The *Δ* *E* values obtained showed that there was no colour change between the two time periods in enamel. However, the *Δ* *E* values were highest for chlorhexidine in 7 days but were highest for Listerine in 15 days. Favaro et al. in their study found the highest colour variation in the Listerine whitening group. All the mouth rinses tested in their study showed a significant colour change. The change in colour in the above study may be directly dependent on the duration of usage.

One important limitation of our study is that the medium in which tooth samples were stored, particularly distilled water, does not accurately reflect the complex physiological conditions present in the oral cavity. Saliva within the mouth contains various minerals, enzymes and organic components that play a significant role in tooth remineralisation and other biological processes, which cannot be simulated by storage in distilled water. This limitation suggests that future research should consider the use of more physiologically relevant storage media, such as artificial saliva, to better mimic intraoral conditions and provide more clinically applicable data.

The main drawback of this study is that it is an in vitro study. The effects observed in in vitro studies are magnified owing to the lack of clinical confounding agents which inhibit the effect of the erosive agents. These include the salivary phosphate and bicarbonate buffers, constant cleaning of the surface with salivary mucin, water and antimicrobial agents as well as the physical cleansing activity of the tongue and mucosa.

It would have been more apropos to evaluate the microhardness and roughness of the teeth within the oral cavity in a paired before and after mouth wash use as a clinical trial. However, a key limitation of designing such a study is the inability to directly measure hardness and surface roughness inside the oral cavity using current standard laboratory‐based methods. Traditional microhardness and roughness tests require specimens to be highly polished, flat and accessible for precise measurements, making them technically challenging or impractical for intraoral assessment in situ. Future prospects should include the development and validation of noninvasive, real‐time intraoral measurement techniques capable of assessing changes in hardness and surface roughness of dental tissues and restorative materials in situ. Innovations such as portable microhardness probes or advanced imaging/laser‐based devices could enhance our ability to assess these important physical properties intraorally, improving both research quality and clinical evaluation.

## 5. Conclusion

Based on the results of this study, we can conclude that mouth rinses can be used without causing visible morphological changes to enamel and cementum for a period of 15 days when used according to the manufacturer’s instructions. However, since they do possess an acidic pH, they do have the ability to alter the surface microstructure of enamel and cementum. It is thus advisable to avoid the use of these mouth rinses prior to brushing or following another erosive challenge, for example, after a carbonated drink. [9] Chlorhexidine, when prescribed by dentists, is usually for short to medium‐term use. However, due to the easy availability of these products over the counter, the dentist must educate the patient to prevent overuse after the prescribed period to avoid any undesirable effects.

## Conflicts of Interest

The authors declare no conflicts of interest.

## Funding

This work was supported by the ICMR STS grant, Project Reference No. 2022‐04005.

## Data Availability

The data that support the findings of this study are available from the corresponding author upon request.
